# Impact of enhanced leaf model on dose calculation accuracy in single‐isocenter multitarget stereotactic radiosurgery treatments

**DOI:** 10.1002/acm2.70039

**Published:** 2025-02-19

**Authors:** Hem Moktan, Hongyu Jiang, H. Harold Li, Kenny Guida

**Affiliations:** ^1^ Department of Radiation Oncology University of Kansas Cancer Center Kansas City Kansas USA; ^2^ Department of Radiation Oncology University of Nebraska Medical Center Omaha Nebraska USA

**Keywords:** enhanced leaf model, MLC modeling, treatment planning, Varian Eclipse v18.0

## Abstract

**Purpose:**

Single‐isocenter multitarget (SIMT) radiosurgery has become increasingly popular as advancement in planning and delivery systems have made this approach clinically viable. With targets varying in size and distance from isocenter, SIMT plans are highly complex with dynamic multileaf collimator (MLC) motion. Our department recently commissioned Eclipse Treatment Planning System v18.0, which included a novel enhanced leaf model (ELM) for photon dose calculation. ELM represents the biggest update in MLC modeling on a commercial treatment planning system over the past decade, yielding improvements in leaf modeling and ray tracing. Considering its dependence on dynamic MLC movements, we set out to assess the potential clinical impact of ELM on SIMT.

**Methods:**

Dynamic zebra crosswalk (DZC) plans were delivered on a Varian Edge to investigate ELM. DZCs consisted of sweeping MLC gaps (ranging 1–15 mm) across a 3 cm width at isocenter, 4 cm, 8 cm, and 12 cm along the x‐axis. Phantom dose calculations were performed using AAA v15.6 and v18.0 (with ELM) for 6 MV flattening filter free DZC plans and compared to measurements using stereotactic radiosurgery MapCHECK (Sun Nuclear Corporation) and Gafchromic EBT4 films (Ashland). To assess potential impact on SIMT, ten patients were retrospectively planned with RapidArc and HyperArc. Both versions of AAA were used for dose calculation.

**Results:**

DZC measurements showed improved agreement with ELM; differences between measured and calculated doses were reduced by as much as 19% for the smallest sweeping gaps at off‐axis distances. Differences in central profile dose for DZCs increased with reduced gap size and increased off‐axis position. SIMT plans showed up to 4.0% increase in planning target volume (PTV) maximum dose when switching from AAA v15.6 to v18.0.

**Conclusion:**

Dose calculations with ELM mirrored diode and film measurements for highly modulated SIMT plans. ELM represents a major improvement in MLC modeling that more accurately reflects current treatment delivery practice.

## 1 INTRODUCTION

Whole brain radiation therapy (WBRT) has historically been the treatment of choice for multiple intracranial metastases.^1^ With the advancement in radiation delivery systems and therapeutic techniques, stereotactic radiosurgery (SRS) has evolved as an alternative to WBRT to achieve intracranial disease control with superior normal tissue sparing and less cognitive deterioration.^2^ Conventional use of SRS in the management of multiple metastases with one or more isocenters is, however, very time‐consuming due to long treatment times affecting patient throughput. Therefore, single‐isocenter multitarget (SIMT) SRS has been adopted as a safe and effective modality for the treatment of multiple brain metastases.^3^ For such treatments, linac‐based volumetric modulated arc therapy (VMAT) SRS has become increasingly popular.^4^ However, the use of VMAT plan optimization for SIMT treatments with targets varying in size and distance from the isocenter essentially makes these plans highly complex.^5^, ^6^

MLC‐shaped small fields used in SRS and SIMT SRS treatments present significant challenges for accurate dose estimation by treatment planning systems (TPSs). One of the studies conducted on TPS dose estimation for very small fields shaped by high‐definition multileaf collimators (HD‐MLC) revealed significant (more than 11%) deviations from film measurements.^7^ Furthermore, another study reported good agreement for larger MLC fields, yet presented substantial inaccuracies for very small fields.^8^ This prompted the authors to recommend caution with RapidArc VMAT plans because these plans may contain error‐prone small subfields.^8^ For MLC shaped fields, MLC modeling in the TPS is one of the most important factors affecting the accuracy of estimated dose. As VMAT can continuously modulate dose rate, gantry speed, and MLC movement simultaneously, accurate representation of the dose delivered in such plans requires precise modeling of MLCs in the dose calculation algorithms used by TPS.^9^

The dosimetric leaf gap (DLG) was originally conceived for MLC modeling in Varian Eclipse TPS (Varian Medical Systems, Palo Alto, CA, USA).^10^ After the introduction of RapidArc VMAT, the tongue‐and‐groove effect was introduced into the dose calculation algorithms to account for chaotic leaf motions with repetitive back‐and‐forth trajectories, often for individual leaves.^10^ This led to the design and implementation of DLG‐based MLC model in Varian Eclipse TPS. In this model, the MLC is viewed as a thin, homogeneous attenuating layer with a constant transmission factor (Tr) over the whole beam. The fluence representation of the MLC gap is also modeled the same, regardless of the position along the X‐axis. The only user‐definable parameter, DLG, models the mechanical MLC offset as well as the penumbra shift due to the rounded leaf tips.^10^ Several studies have reported drawbacks of this model producing unsatisfactory agreement between measured and calculated doses; this necessitated the tuning of the DLG parameter to minimize the differences and demonstrated the need for an improved MLC model for high‐precision SRS and SBRT treatments.^11^, ^12^, ^13^, ^14^

The enhanced leaf model (ELM) was introduced in Eclipse v18.0 in an effort to improve MLC modeling for an era in radiation oncology dominated by the use of VMAT. In this model, the MLC is no longer considered as a homogeneous, thin attenuating layer; rather, ELM aims to model the true shape of the MLCs, where the attenuation model is based on the leaf design.^15^ Contrary to the DLG‐based MLC model, the MLC leaf tip shape, drive screw cutout, and leaf body thickness are modeled using divergent ray tracing through the leaf along the leaf axis direction. The definition of Tr has been revised, making it an MLC type and beam energy dependent factor.^15^ This has been shown to provide better fluence estimation for off‐axis positions. The leaf gap (LG) parameter no longer includes the effect of rounded leaf tip but represents the true mechanical calibration and is a treatment unit‐specific parameter.^15^

Our department recently commissioned Eclipse v18.0 with ELM for HD‐MLC and Millennium MLC models for photon dose calculation algorithms following AAPM MPPG 5a.^16^ The differences in dose prediction for MLC‐based fields comparing ELM to DLG‐based MLC models have been demonstrated by Van Esch et al.^15^ The improved prediction accuracy by ELM was verified through experimental measurements on several test plans involving static and dynamic MLC strips.^15^ However, these test plans do not represent the actual clinical SIMT VMAT plans, which are significantly more complex. Therefore, this study aimed to investigate clinical impact of ELM on SIMT treatments, which has not yet been performed.

This study conducted a thorough investigation of dose calculation with ELM for dynamic MLC strips of varying MLC sweeping gap widths at different off‐axis locations and compared them to those calculated by DLG‐based MLC model. Experimental measurements were carried out to delineate the accuracy of small field dose estimation by ELM and quantify discrepancies between ELM‐ and DLG‐based calculations. Furthermore, the impact of MLC modeling on highly modulated RapidArc and HyperArc SIMT plans was also investigated. The calculated doses on PTVs of varying sizes and at different off‐axis locations were assessed. QA measurements were also performed on selected targets to validate the TPS dose calculation accuracy.

## 2 METHODS

### 2.1 MLC parameter configuration for ELM

To configure ELM in Eclipse v18.0, measurements similar to those proposed by LoSasso et al. were performed.^10^, ^15^ The transmission measurements were identical to DLG‐based measurements to assign the average Tr. Three sweeping gaps of 4, 6, and 20 mm in a 10 × 10 cm^2^ collimated field were used for LG measurements. The transmission and sweeping gap measurements were performed on a TrueBeam Edge equipped with HD‐MLC. A solid‐water phantom with measurement depth of 10 cm and SSD of 90 cm was used. A 0.6 cc ionization chamber was used to assure adequate averaging over inter‐ and intra‐leaf transmission. Raw measurement data was imported into the beam configuration workspace within ARIA. The beam configuration software fit the LG parameter iteratively to provide optimal agreement between measured and ELM‐calculated doses for a selection of sweeping gap deliveries.^15^

### 2.2 Dynamic zebra crosswalk

Similar to Van Esch et al., Dynamic Zebra Crosswalk (DZC) plans were investigated.^15^ In this case, sweeping MLC gaps of 1, 2, 3, 5, 10, and 15 mm across a 3 cm‐wide field were generated; the sweeping gaps were centered around the central beam axis and at 4, 8, and 12 cm off‐axis locations. All DZC fields were calculated on a 30 × 30 × 30 cm^3^ water phantom in Eclipse, with SSD set to 90 cm. 6‐MV flattening‐filter‐free (6X‐FFF) beams were used for DZC. Final dose calculations were performed using Analytical Anisotropic Algorithm v18.0 (ELM‐based) and v15.6 (DLG‐based) with the dose grid size of 0.125 cm. The lateral cross‐plane calculated dose profiles at the depth of 10 cm were compared between both AAA versions. To analyze differences in the calculated dose profiles, both percent difference at the center of the dose profile and 1D gamma analysis were utilized. Nelms et al. suggested that using more sensitive metrics and tight tolerances during commissioning processes could be informative and highlight potential systematic errors.^17^ The percent differences in dose at the center of the profile were used to quantify the differences in dose calculation by ELM and DLG‐based MLC models. 1D absolute gamma analysis, using 2%/2 mm criteria and 10% dose threshold, was performed comparing dose calculations with AAA v18.0 and v15.6 at a depth of 10 cm for each DZC gap.

To investigate the accuracy of dose prediction by AAA v18.0, the experimental measurements of DZC were performed on a TrueBeam Edge using a Sun Nuclear (SNC) (Melbourne, FL, USA) SRS MapCHECK diode array detector. For all DZC measurements, the diode detector plane was set to a source‐detector distance of 100 cm; solid water slabs were added to achieve a 10 cm equivalent depth to match the TPS generated plan. Due to a limited field size of 7.7 × 7.7 cm^2^, the SRS MapCHECK was shifted and centered accordingly for all the off‐axis DZC measurements. The experimental cross‐plane dose profiles were then compared to the calculated dose profiles to assess the differences between calculated and measured doses. Percent differences at the center of the profile between experimental measurements and TPS calculations were evaluated to validate differences between the dose calculation algorithms. 1D absolute gamma analysis, using 2%/2 mm criteria and 10% dose threshold, was also performed, comparing measurements with AAA v18.0 and v15.6 dose calculations.

Gafchromic EBT4 film (Ashland Inc, Bridgewater, NJ, USA) measurements were also utilized for absolute dose measurements for the test plans to supplement diode measurements. Film measurements were deemed ideal as studies have shown that dose response for EBT4 is independent of energy for photon beams in the MV range.^18^ The DZC measurements were performed with the same setup as diode measurements. The films were scanned using EPSON EXPRESSION 10000 XL professional grade film scanner after the film developments were stabilized. Red channel readings were used to calculate the optical density and, subsequently, measured dose.

### 2.3 RapidArc and HyperArc SIMT VMAT plans

To investigate the clinical impact of ELM on dosimetric accuracy and plan quality for SIMT treatments, ten patients were retrospectively replanned with RapidArc and HyperArc VMAT using 6X‐FFF beam energy. Plans were optimized using Photon Optimizer (PO) v15.6. The optimization was pushed to achieve desired prescription dose coverage to the PTVs and sharp dose fall‐off beyond the PTV for both delivery techniques; all PTVs were optimized to deliver a V100% of 100% with a weight of 150. HyperArc plans utilized an automated SRS NTO (weight 150) to achieve desired dose falloff. RapidArc optimization relied on a manual NTO setting (weight 150, 0.1 cm from target, 99% starting dose, 30% ending dose, 0.3 falloff) and in‐house Knowledge‐Based Planning modeling to reduce OAR dose. Additional optimization settings included enabling convergence mode and setting the aperture shape controller to the low setting. After optimization, the final dose calculations were performed using both AAA v18.0 and v15.6 with the dose grid size of 0.125 cm, keeping control points and monitor units identical for each plan type.

A total of 89 PTVs (range: 3–16) across all the SIMT plans were analyzed in this study. PTV volumes ranged from 0.04 cc to 17.29 cc. The PTV max point doses (*D*_max_) in all the PTVs calculated by AAA v18.0 were compared to those calculated by AAA v15.6. Maximum displacements along the x‐axis were tabulated for all PTVs across all arcs. Prescribed dose to each lesion ranged from 15–20 Gy. Paired *t*‐tests were used to assess significance between the dose calculated to each PTV with AAA v15.6 and v18.0.

To evaluate the accuracy of MLC modeling on dose calculation, QA plans were generated and a true composite fluence was delivered on SNC SRS MapCHECK with StereoPHAN head phantom. The SRS MapCHECK detector plane was rotated to a desired angle to align PTV dose distributions on the detector plane. The measured dose was then compared to the TPS calculated dose using 2D absolute gamma analysis. To focus on the high‐dose region, a gamma criterion of 0%/1 mm with a dose threshold of 50% of the global maximum was used to perform gamma analysis for both SRS VMAT and HyperArc plans. It is important to evaluate the gamma pass rates in the high‐dose volumes, as others have reported that failing points within the tumor volumes can be masked by the low‐dose regions within the normal brain.^19^

## 3 RESULTS

### 3.1 ELM measurements

Using the fitting provided by the v18.0 Beam Configuration workspace, the 6X‐FFF AAA v18.0 ELM LG parameter was determined to be −0.0170 cm; comparatively, the DLG measured for 6X‐FFF was 0.0709 cm. The ELM LG parameters are negative as opposed to positive DLG values. This parameter represents the physical separation between MLC leaves in opposing banks and, therefore, is a representation of mechanical calibration. MLC transmission for 6X‐FFF was measured as 0.0106. The LG for the Edge was set to 0.04 cm during commissioning, and the center offsets for MLC banks A and B were 0.06 and 0.02 cm, respectively. The ELM LG parameters are treatment unit‐specific, as mechanical calibration may differ amongst treatment units.^15^

### 3.2 Evaluation of DZC

**FIGURE 1:**
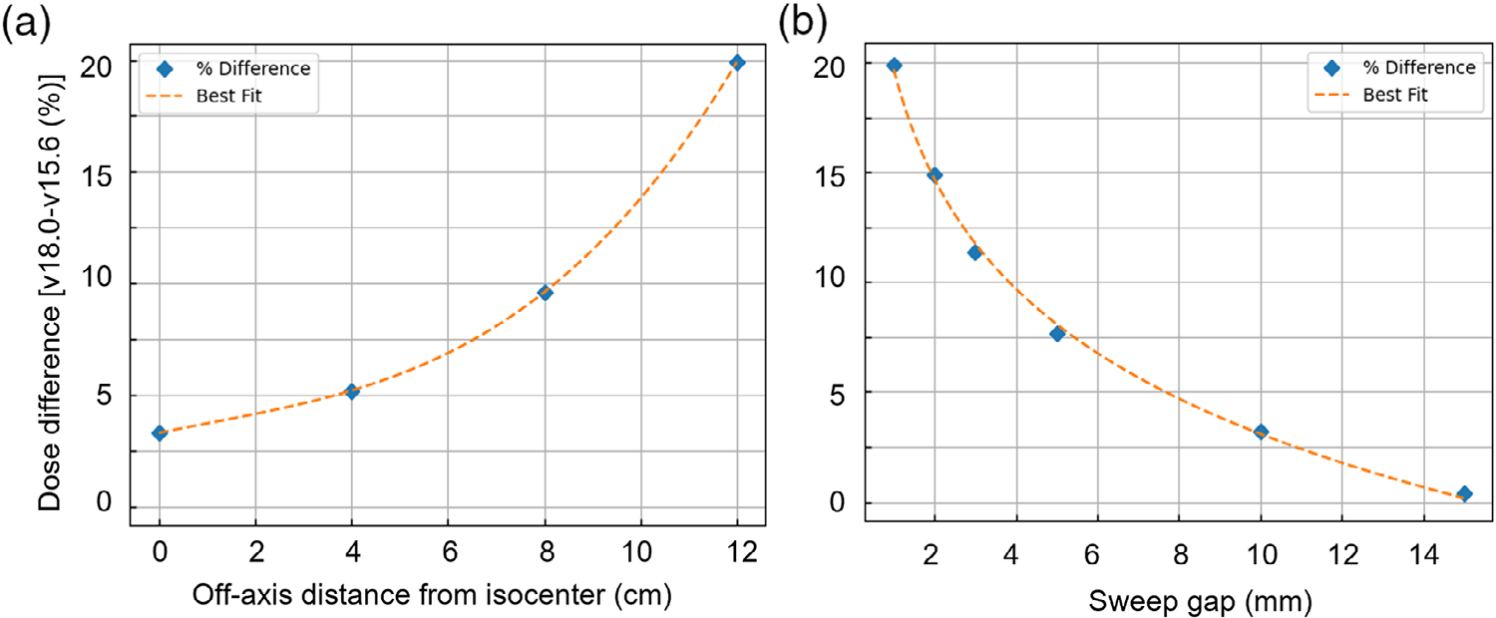
(a)–(b) The DZC profile central dose difference (%) plotted against (a) off‐axis distance from the isocenter for 1  mm sweeping gap width and (b) MLC sweeping gap width for DZC at 12 cm off axis distance. DZC, dynamic zebra crosswalk.

The percentage differences in DZC central profile doses between AAA v18.0 and v15.6 are tabulated in Table 1. The impact of the MLC model was found to be more pronounced for smaller sweeping gaps at distances further off‐axis; for a 1 mm sweeping gap width at 12 cm off‐axis distance, the difference was as high as 19%. Furthermore, the difference was found to decrease with increasing MLC sweeping gap width and increase with increasing off‐axis distances, as shown in Figure 1a,b. Similarly, 1D absolute gamma analysis with 2%/2 mm criteria and 10% threshold shows reduced agreement between v18.0 and v15.6 dose calculations for both smaller gap sizes and increased off‐axis distances. For gap size of 3  mm and greater, gamma pass rates at isocenter were 90% or greater when comparing v15.6 calculations to v18.0. For 1–2 mm gaps, gamma pass rates dropped below 75% at all off‐axis distances; gamma pass rates decreased as off‐axis distance increased from 0 to 12 cm, as evidenced in rates of 48.2% and 59.8% at 12 cm off‐axis distances for 1 and 2 mm sweeping gaps, respectively.

**TABLE 1:** Central profile dose difference (%)/gamma pass rates (%) for DZC profiles calculated using AAA v18.0 and AAA v15.6 for 6X‐FFF beam at different off‐axis distances with varied sweeping gaps.

	Sweeping gap (mm)					
Off‐axis distance (cm)	1	2	3	5	10	15
0	3.3/53.6	2.3/74.2	1.8/92.1	1.3/100.0	0.7/100.0	0.4/96.5
4	5.2/54.8	3.7/75.0	3.0/78.8	2.0/92.7	1.1/100.0	0.0/98.8
8	9.6/58.6	6.9/69.8	5.4/70.3	4.0/68.8	1.8/100.0	0.3/100.0
12	19.9/48.2	14.9/59.8	11.4/62.4	7.7/58.7	3.2/81.4	0.4/100.0

**FIGURE 2:**
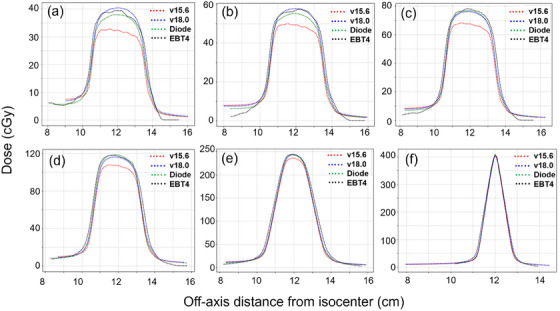
Crossline dose profiles of DZC for different sweeping gap widths of (a) 1 mm, (b) 2 mm, (c) 3 mm, (d) 5 mm, (e) 10 mm, and (f) 15 mm at 12 cm off‐axis distance estimated with AAA v15.6 and v18.0 and measured with SNC SRS MapCHECK diode array (Diode) and Gafchromic EBT4 film. DZC, dynamic zebra crosswalk; SNC, Sun Nuclear; SRS, stereotactic radiosurgery.

The DZC cross‐plane dose profiles calculated with AAA v15.6 and v18.0 were compared to profiles measured with the SRS MapCHECK and EBT4 film. Figure 2 shows all sweeping gap widths at an extreme 12 cm off‐axis distance. The DZC profiles calculated with ELM matched very well, with the measured profiles with maximum difference of 2.2% seen amongst these comparisons. However, differences between DLG‐based AAA v15.6 calculations differed greatly with SNC and EBT4 at 12 cm off‐axis, with percent differences seen as high as 19%. The differences in the profile amplitude were apparent for smaller sweeping gap widths; however, the effects were found to decrease for increased sweeping gap widths, as shown in Figure 2.

Tables 2 and 3 showcase the differences between measured DZC central profiles and AAA v18.0 and v15.6, respectively. Central profile dose differences and gamma pass rates with ELM agreed very well with the experimental measurements, as shown in Table 2. However, central profile doses based on the DLG model were found to be smaller than the measured values in all scenarios, leading to poor gamma pass rates, as shown in Table 3. Differences were significant for small sweeping gap widths at larger off‐axis distances; for 1 mm gaps at 12 cm off‐axis, AAA v18.0 calculated dose was within 0.6% of diode array measured data, while that of AAA v15.6 was 19% lower, leading to a gamma pass rate of 34.2% (using 2%/2 mm criteria), as compared to the 89.1% pass rate seen with the v18.0 comparison. Furthermore, film measurements performed for DZC dose for different sweeping gap widths at 12 cm off‐axis distances corroborated the diode measurements within reasonable uncertainties, as shown in Tables 2 and 3. Therefore, DZCs showed improved dose calculation with ELM, which was consistent with the findings demonstrated by Table 1 and Figure 1.

**TABLE 2:** Central profile dose difference (%)/gamma pass rates (%) for DZC profile calculated using AAA v18.0 relative to those measured using SNC SRS MapCHECK and EBT4 film (only for 12  cm off‐axis distance) for 6X‐FFF beam.

	Sweeping gap (mm)					
Off‐axis distance (cm)	1	2	3	5	10	15
0	1.4/100.0	3.6/64.7	2.9/70.4	2.8/56.9	1.0/100.0	2.5/96.5
4	1.5/100.0	1.0/100.0	2.0/96.8	2.8/96.8	1.1/100.0	2.2/98.8
8	1.3/100.0	0.8/100.0	2.7/91.0	1.7/97.5	0.1/100.0	2.0/100.0
12	0.6/89.1	1.3/100.0	1.9/100.0	2.2/86.7	0.3/100.0	1.4/100.0
12 (Film)	2.4/56.2	1.8/100.0	2.5/90.3	1.0/71.6	0.3/100.0	0.2/100.0

**TABLE 3:** Central profile dose difference (%)/gamma pass rates (%) for DZC profiles calculated using AAA v15.6 relative to those measured using SNC SRS MapCHECK and EBT4 film (only for 12  cm off‐axis distance) for 6X‐FFF beam.

	Sweeping gap (mm)					
Off‐axis distance (cm)	1	2	3	5	10	15
0	1.9/64.0	5.8/59.0	4.7/57.4	4.0/53.2	1.7/100.0	2.1/93.9
4	3.9/34.0	4.7/58.2	4.9/65.7	4.7/65.7	2.2/90.5	2.2/98.8
8	8.4/28.5	7.6/40.3	7.6/56.4	5.6/56.4	1.8/100.0	2.2/99.3
12	19.4/34.2	15.4/36.7	13.0/53.1	9.7/53.1	2.9/82.8	1.8/100.0
12 (Film)	17.9/18.3	12.9/37.1	13.6/39.3	8.6/44.8	3.5/80.7	0.7/100.0

### 3.3 SIMT VMAT plan quality and PTV dose evaluation

**FIGURE 3:**
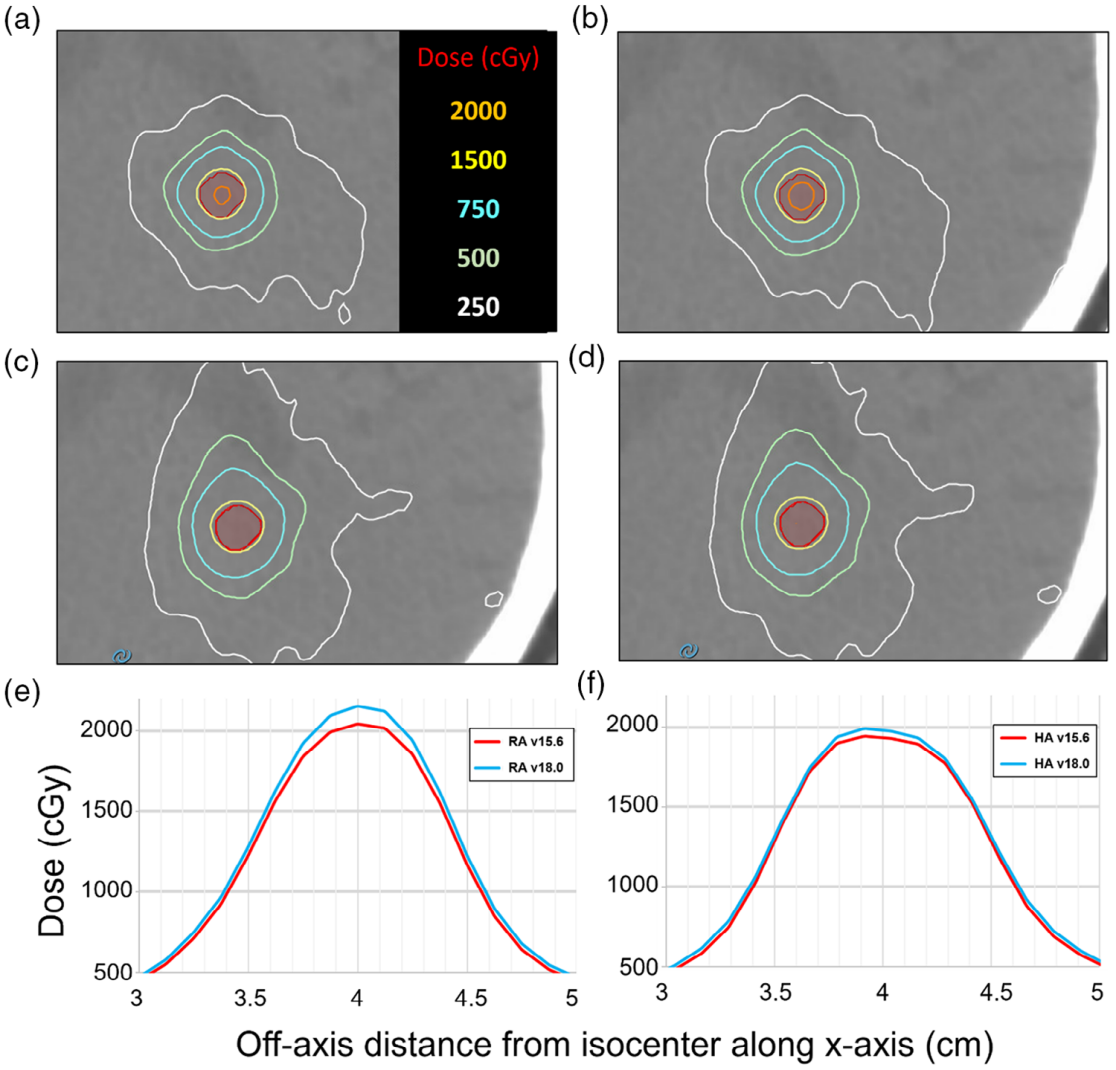
(a)–(f) Isodose lines for RapidArc (RA) (v15.6 – a, v18.0 – b) and Hyperarc (HA) (v15.6 – c, v18.0 – d) for a 0.1cc PTV (red) with a maximum distance of 4.2  cm along the x‐axis. Dose profiles across the lesion and surrounding normal brain (e–f) show that the differences are pronounced within the high‐dose region for both RapidArc and HyperArc, respectively. This small lesion showed an increase in dose to the PTV of 4% for RapidArc and 2.6% for HyperArc when recalculating with AAA v18.0 with ELM. ELM, enhanced leaf model.

All SIMT treatment plans evaluated in this study were very similar in terms of target coverage with comparable conformity indices and gradient measures. A deeper look into PTV dose revealed hotter PTV cores with AAA v18.0 as compared to AAA v15.6, as shown in Figure 3a–f.

**FIGURE 4:**
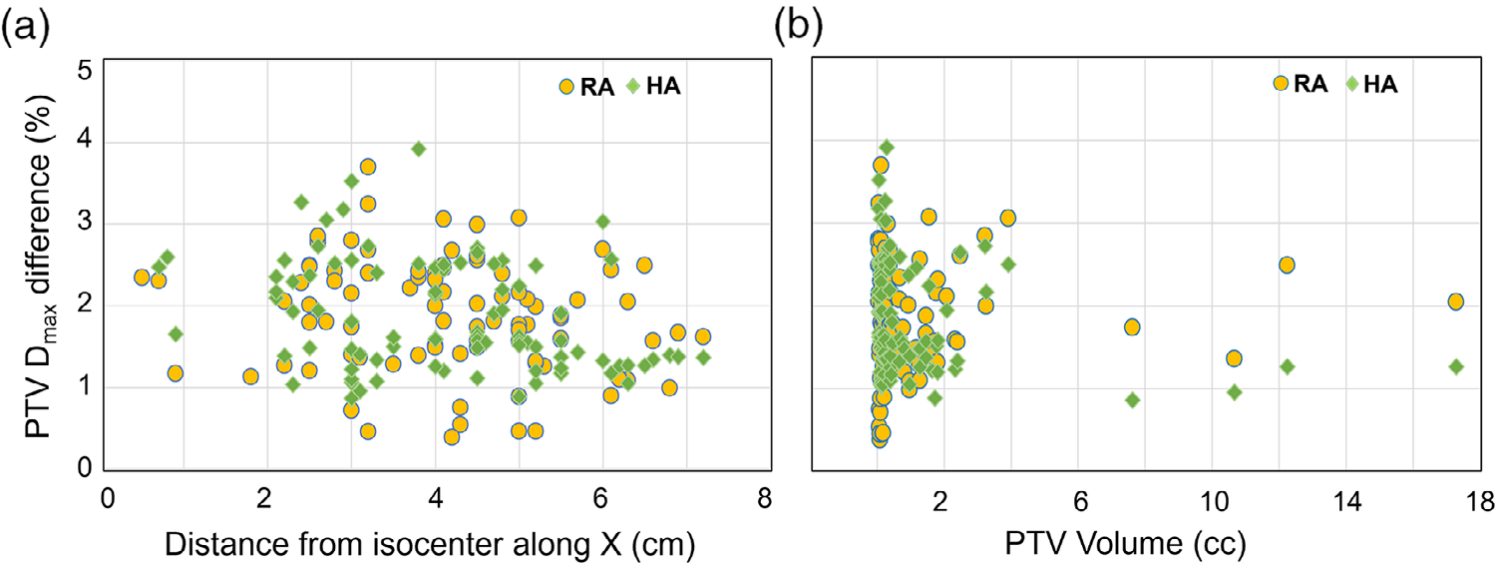
(a)–(b) The percent difference in *D*_max_ for evaluated PTVs calculated using AAA v18.0 compared to that of v15.6 in RapidArc VMAT (RA) and HyperArc VMAT (HA) (a) plotted against the PTV off‐axis distance from isocenter along x‐direction, and (b) plotted against PTV volume. *D*_max_, max point dose. VMAT, volumetric modulated arc therapy.

To evaluate the effect of MLC models in the calculated dose, percent differences in PTV *D*_max_ calculated by both versions of AAA were analyzed. Dose distributions were notably hotter in the PTV regions during plan comparison; changes within normal brain dose were negligible across the 10‐patient cohort. There was a significant increase (*p*‐value < 0.05) in PTV *D*_max_ when switching from AAA v15.6 to 18.0, with average percent differences found to be 1.9% (range: 0.4%–3.7%) for RapidArc, and 1.9% (range: 0.9%–3.9%) for HyperArc VMAT. Percent differences were plotted against PTV maximum off‐axis distance along the x‐axis and PTV volumes, as shown in Figure 4a,b. The differences were found to be higher for PTVs at a maximum displacement of 3–5 cm from isocenter along the x‐axis, as shown in Figure 4a. Smaller PTVs were found to have higher *D*_max_ differences in comparison to the larger PTVs, as seen in Figure 4b. The maximum differences seen amongst the lesions were about 4%, which are clinically significant.

### 3.4 Patient‐specific QA for SIMT VMAT

**FIGURE 5:**
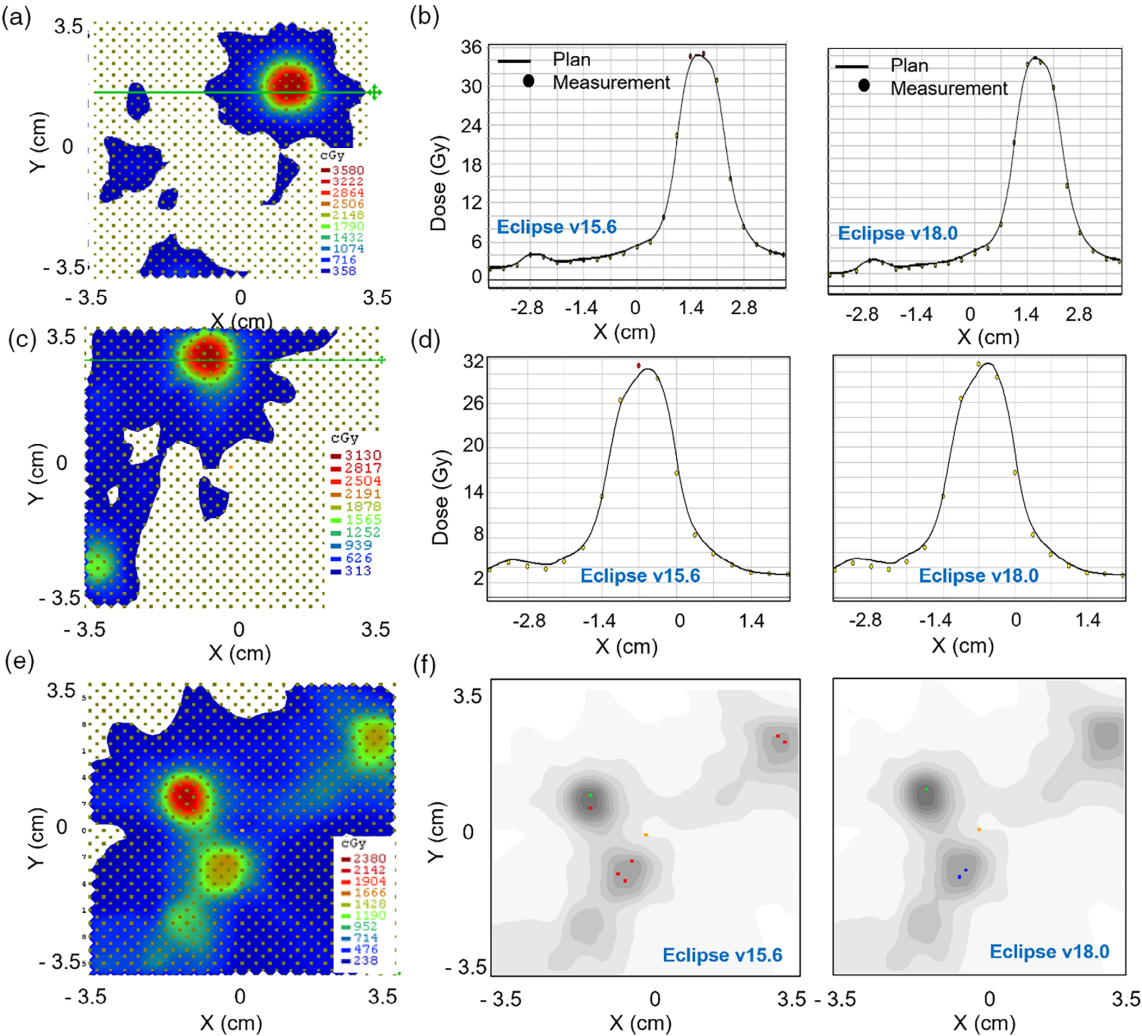
(a)–(f) PSQA measurements performed for lesions at 1.8 cm (a–b), 2.9 cm (c–d), and 5.0 cm (e)–(f) off‐axis from isocenter along the x‐direction. Measured dose plans are provided in (a), (c), and (e), with corresponding dose profile comparisons (b) and (d) between measured dose and AAA v15.6 and v18.0 dose calculations; in the case of (e)–(f), a grayscale gamma comparison between measured dose and calculations via v15.6 and v18.0 was provided to show a comparison between failing points. Using gamma criteria of 0%/1 mm, passing rates increased for all three cases when switching from AAA v15.6 to v18.0; cases (a), (c), and (e) improved from 85.7% to100%, 93% to 100%, and 87.9% to 92.5%, respectively. PSQA, patient‐specific QA.

To further evaluate the effect of MLC modeling on SIMT, patient‐specific QA (PSQA) measurements were performed for selected PTVs. The measurements and gamma comparisons for three PTVs at different off‐axis distances are displayed in Figure 5a–f. The PSQA measurements were compared to TPS calculated dose through gamma analysis. Using departmental standards of gamma analysis (2%/1 mm, 10% dose threshold), both plans exceeded 95% pass rates; reducing gamma criteria to 1%/1 mm yielded similar results. However, the v15.6 plan prediction was noticeably colder along dose profiles in the high‐dose regions for off‐axis PTVs. For a PTV (0.5 cc) at 1.8 cm off‐axis location along the x‐direction (Figure 5a,b), the gamma passing rate (0%/1 mm, 50% dose threshold) for AAA v18.0 was 100%, whereas the passing rate dropped to 85.7% for AAA v15.6. Using the same criteria, the gamma passing rates for another plan (Figure 5c,d) involving a PTV (0.7 cc) with a maximum displacement of 2.9 cm were 93% and 100% for AAA v15.6 and v18.0, respectively. In this case, gamma analysis identified two hot failing points (Figure 5c) when comparing the measured dose to v15.6 calculation within the PTV, whereas these points pass when compared to v18.0 calculation. Shifting the SRS MapCHECK allowed for measuring targets at greater displacements from isocenter. For a case with a 0.7 cc lesion (Figure 5e,f) with a maximum displacement of 5 cm along the x‐axis and maximum dose difference of over 3% between v15.6 and v18.0 calculations in Eclipse, QA measurements showed a noticeable improvement with v18.0, yielding a passing rate of 92.5% versus 87.9% with better agreement in the high‐dose region for both the PTV and two nearby lesions. This concluded that the differences in dose calculation by AAA v18.0 as compared to AAA v15.6 in the PTVs at larger off‐axis distance from the isocenter were more significant.

The failing points for these cases calculated with AAA v15.6 were found to be in the high‐dose regions of the PTV. From these measurements, it is apparent that the DLG‐based MLC model in AAA v15.6 underestimated the dose in high‐dose regions within the PTV, seen in Figure 5b,d,f. Measured dose was found to agree very well with AAA v18.0 with ELM, with these plans yielding higher gamma passing rates with fewer failing points in high‐dose regions of the PTV.

## 4 DISCUSSION

The primary goal of this study was to investigate the potential clinical impact of ELM, a recent development in MLC modeling introduced in Eclipse v18.0. Some of the tests in this study were based on the previous work by Van Esch et al., where they demonstrated improved accuracy with ELM models compared to DLG‐based MLC models for both TrueBeam and Halycon LINACs.^15^ Van Esch demonstrated improvements in dose calculation for modulated fields both at isocenter and at off‐axis positions. Similar to our results, Van Esch measured 3%, 4%, 6%, and 12% differences between ELM and DLG calculation models at 0, 4, 8, and 12 cm off‐axis for a 3 mm DZC on a TrueBeam. These results led us to conduct our own series of tests to confirm these differences between v15.6 and v18.0 calculations and see how differences in MLC modeling could affect dose calculation for cases involving small lesions targeted by dynamic MLC movements. Our study confirmed that dose calculations utilizing ELM more accurately represent the measured dose delivered in both simple test plans and complex SIMT VMAT plans.

In DZC plans, the differences in dose calculation between AAA v18.0 with ELM and AAA v15.6 with DLG were found to be dosimetrically significant for smaller sweeping gap widths at larger off‐axis distances. This was further confirmed and validated with the experimental measurements by diode array detectors and Gafchromic films. These findings were in accordance with the previous study, confirming improved correlation between calculated and delivered DZC dose profiles with ELM.^15^ For the 3 mm DZC plans, v15.6 calculations differed from diode array measurements by 4.9%, 7.6%, and 13.0% at off‐axis positions of 4, 8, and 12 cm, whereas v18.0 calculated dose had a maximum difference of 2.7% from measured dose off‐axis; 1D gamma comparisons corroborated these results. The DZC plans with small fields both at isocenter and off‐axis were designed to assess the impact of ELM on both IMRT and VMAT plans using small MLC openings.^15^ Using the range of gaps from 1  mm to 15 mm was important, as these sizes span typical sizes of tumors treated clinically with SIMT. For SRS treatments, tumor volumes can range drastically; cases used in this study targeted lesions as small as 0.04 cc, which necessitate the use of small MLC openings to deliver prescription dose while maintaining desired levels of conformity and normal tissue sparing. The chaotic nature of MLC movements in SIMT cases introduce small MLC openings, so it was important to identify differences in dose calculation for very small sweeping gaps.

Traditionally, our clinic refrains from treating lesions outside of a 6 cm sphere for SIMT cases; if a case presents an array of lesions throughout the brain, multiple isocenters are typically used to mitigate the impact of rotational error at larger distances from isocenter. Despite this, we still examined the impact of DZC at large distances off‐axis due to the acceptance of treating SIMT with a single isocenter at other institutions.^20^, ^21^

For all the SIMT VMAT plans evaluated in this study, the differences were not as apparent as to those observed in simple DZC plans. With SIMT being delivered via VMAT, target off‐axis distances vary throughout each arc length, with MLCs continuously moving to shape dose distributions. The impact of off‐axis delivery can be blurred out by the nature of VMAT and arc geometry, but it is worth noting that differences were seen to increase for targets that were 3–5 cm off‐axis in the x‐direction during the course of treatment. *D*_max_ differences between ELM and DLG‐based algorithms were found to plateau beyond maximum off‐axis distances of 5 cm, as evidenced in Figure 4a. We hypothesize that the differences were minimized due to averaging effect from multiple arcs delivery, each with different collimator angles and dynamic MLC movements. Tumor location could also influence these values, as lesions closely grouped together could lead to larger MLC openings. Consequently, VMAT plans covering large PTVs with large MLC openings tended to have minimal differences, whereas the effects were more pronounced for smaller PTVs at larger off axis distances, as shown in this study.

We planned SIMT with RapidArc and HyperArc VMAT, as our department employs both delivery systems clinically. We noticed that differences between v15.6 and v18.0 were very similar between RapidArc and HyperArc VMAT plans for 6X‐FFF, suggesting that VMAT optimization and delivery technique has minimal impact on the differences between ELM and DLG modeling.

It is worth noting that SIMT treatments often involve very small targets at extreme off‐axis distances and therefore performing dose calculations with a more accurate MLC model can ensure improved treatment plan quality and increased accuracy of delivered dose. We did see an increase in dose to every target across the entire cohort of plans when ELM was implemented; dose to normal brain tissue remained relatively unchanged. Van Esch et al. suggested that dose calculations using ELM could be similar or higher than dose calculations using DLG for IMRT and VMAT plans.^15^ These differences were seen in the TPS and in SRS MapCHECK measurements, which showed a closer correlation in high‐dose regions between dose calculated with ELM than DLG‐based MLC models. With traditional gamma criteria of 2%/1 mm, we achieved passing rates of over 95% for SIMT plans for each MLC model; even with standard gamma criteria, there is a possibility of false positives and negatives existing within the high‐dose regions on the detector plane.^22^ Despite the high pass rates for off‐axis lesions for DLG‐based calculations, we typically noticed differences between the dose profile and diode measurements within the high‐dose regions. As suggested by Nelms et al., conventional gamma criteria may be insufficient when analyzing PSQA^17^, ^22^; tightening gamma criteria may help identify errors, especially during commissioning processes, as in the case of a new MLC model. By reducing the gamma criteria to 0%/1  mm and focusing on just the high‐dose regions (by changing the threshold dose to 50%), we were able to clearly see differences in how MLC modeling could impact PSQA, with plans calculated with ELM clearly showed improved gamma pass rates for small, off‐axis targets. Similar to Nelms’ findings, we found that using more sensitive metrics, including both maximum dose difference and tighter gamma analysis, were more useful in the commissioning of ELM.^17^ With SIMT delivering 15 Gy or more to multiple lesions within the brain, having an accurate dose calculation is paramount in ensuring safe and accurate delivery. ELM modeling has given our team more confidence in the delivery of SIMT. Improvements in MLC modeling and ray tracing have made ELM the MLC model of choice for dose calculation at our institution.

## 5 CONCLUSION

Dose calculations with ELM were found to correlate closer to measured dose distributions than DLG‐based MLC model for small, dynamic MLC apertures both at isocenter and different off‐axis positions along the x‐axis. The differences were more pronounced for smaller MLC apertures and larger off‐axis distances. ELM also had impact on clinical plans for SIMT VMAT. ELM yielded better agreement between calculated and delivered SIMT VMAT treatment plans, as it demonstrated more accurate dose calculation within the high‐dose regions. Therefore, ELM represents presumably the biggest commercial improvement in MLC modeling since DLG.

## AUTHOR CONTRIBUTIONS

**Hem Moktan**: Lead author; treatment planning; data collection; manuscript review; and preparation. **Hongyu Jiang**: Manuscript review; data collection. **H. Harold Li**: Manuscript review and preparation. **Kenny Guida**: Senior author; treatment planning; data collection; manuscript review; and preparation.

## CONFLICT OF INTEREST STATEMENT

The University of Kansas Cancer Center participated in Varian's Limited Launch Program (LLP) for Eclipse v18.0. Dr Guida reports honoraria from Varian Medical Systems outside of the submitted work.
